# Modulation of *Campylobacter jejuni* adhesion to biotic model surfaces by fungal lectins and protease inhibitors

**DOI:** 10.3389/fcimb.2024.1391758

**Published:** 2024-04-22

**Authors:** Blaž Jug, Maja Šikić Pogačar, Meta Sterniša, Tadeja Tumpej, Katarina Karničar, Dušan Turk, Tomaž Langerholc, Jerica Sabotič, Anja Klančnik

**Affiliations:** ^1^ Department of Food Science and Technology, Biotechnical Faculty, University of Ljubljana, Ljubljana, Slovenia; ^2^ Department of Pediatrics, Faculty of Medicine, University of Maribor, Maribor, Slovenia; ^3^ Department of Biotechnology, Jožef Stefan Institute, Ljubljana, Slovenia; ^4^ Department of Biochemistry and Molecular and Structural Biology, Jožef Stefan Institute, Ljubljana, Slovenia; ^5^ Centre of Excellence for Integrated Approaches in Chemistry and Biology of Proteins, Ljubljana, Slovenia; ^6^ Department of Microbiology, Biochemistry, Molecular Biology and Biotechnology, Faculty of Agriculture and Life Sciences, University of Maribor, Maribor, Slovenia

**Keywords:** *Campylobacter jejuni*, fungal lectins, fungal protease inhibitors, Caco-2 assay, adhesion, invasion

## Abstract

*Campylobacter jejuni*, a Gram-negative bacterium, is one of the most common causes of foodborne illness worldwide. Its adhesion mechanism is mediated by several bacterial factors, including flagellum, protein adhesins, lipooligosaccharides, proteases, and host factors, such as surface glycans on epithelial cells and mucins. Fungal lectins, specialized carbohydrate-binding proteins, can bind to specific glycans on host and bacterial cells and thus influence pathogenesis. In this study, we investigated the effects of fungal lectins and protease inhibitors on the adhesion of *C. jejuni* to model biotic surfaces (mucin, fibronectin, and collagen) and Caco-2 cells as well as the invasion of Caco-2 cells. The lectins *Marasmius oreades* agglutinin (MOA) and *Laccaria bicolor* tectonin 2 (Tec2) showed remarkable efficacy in all experiments. In addition, different pre-incubations of lectins with *C. jejuni* or Caco-2 cells significantly inhibited the ability of *C. jejuni* to adhere to and invade Caco-2 cells, but to varying degrees. Pre-incubation of Caco-2 cells with selected lectins reduced the number of invasive *C. jejuni* cells the most, while simultaneous incubation showed the greatest reduction in adherent *C. jejuni* cells. These results suggest that fungal lectins are a promising tool for the prevention and treatment of *C. jejuni* infections. Furthermore, this study highlights the potential of fungi as a rich reservoir for novel anti-adhesive agents.

## 1 Introduction

*Campylobacter jejuni* is the most common bacterial cause of human gastroenteritis (called campylobacteriosis) worldwide. In the EU, campylobacteriosis is the most commonly reported foodborne disease, with an estimated annual cost of €2.4 billion (ECDC, 2023). Livestock and pets represent reservoirs of *C. jejuni*, which is well adapted and exposed to many antibiotics. Such exposure contributes to its increasing resistance to antibiotics, including those used to treat human diseases (ECDC, 2023), and thus compromises the effectiveness of antibiotic treatments. Thus, research is under way to find new, alternative, non-antibiotic interventions (Dai et al., 2020).

**Figure 1:**
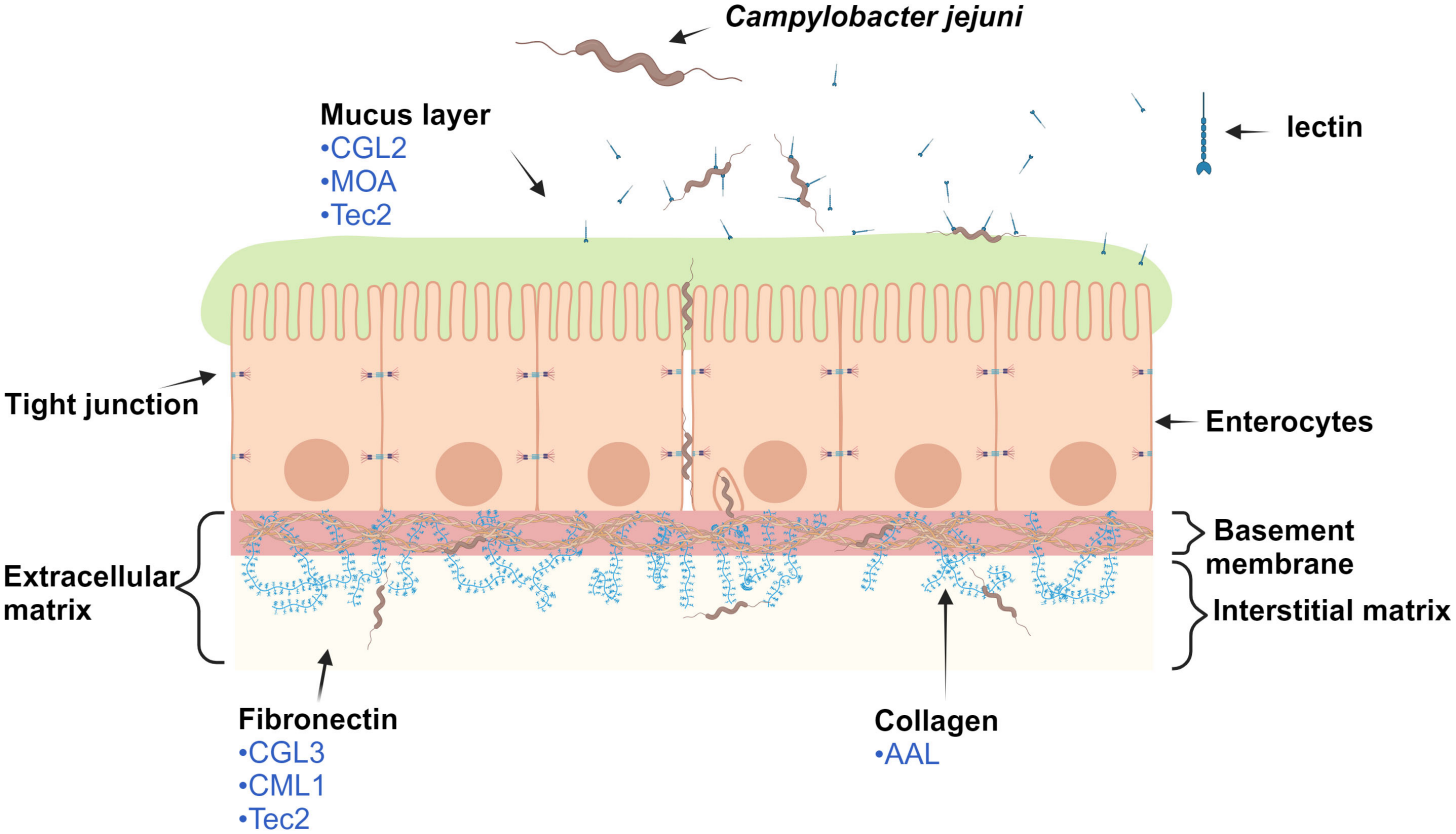
Schematic representation of the monolayer of epithelial cells (enterocytes) and selected macromolecules of the extracellular matrix of the human digestive tract. For clarity, only macromolecules of the extracellular matrix that are involved in paracellular transport and internalization of *Campylobacter jejuni* into the host cell are shown. The lectins (in blue) displayed have been identified by our experiments as the most effective in reducing *C. jejuni* adhesion. Note: elements are not to scale. The image was created using BioRender.com. Abbreviations are defined in **Table 1**.

Protein and glycan interactions between hosts and bacteria play an important role in bacterial adhesion and invasion of the gastrointestinal tract. *C. jejuni* is ingested via contaminated food and enters intestinal crypts lined with mucus (**Figure 1**) containing the glycoprotein mucin. *C. jejuni* is attracted to mucin and metabolizes its components, thereby increasing its own motility and reproduction, which contributes to its successful colonization of intestinal mucosa (Alemka et al., 2012). Upon contact with enterocytes, *C. jejuni* secretes the trypsin-like high temperature requirement A serine protease (HtrA), which cleaves a temporary opening in cell junctions (Boehm et al., 2012). This enables *C. jejuni* to undergo paracellular transport to the basolateral side, which is rich in fibronectin and collagen. Fibronectin is part of the extracellular matrix and a known binding target for the adhesins CadF (*Campylobacter* adhesion to fibronectin) and FlpA (fibronectin-like protein A) of *C. jejuni* (Kuusela et al., 1989). This additional binding to different molecules in the extracellular matrix enables the internalization of *C. jejuni* into host cells (Kovács et al., 2020).

Glycans present on the surface of *C. jejuni* greatly influence its interactions with surfaces (Sabotič et al., 2023). Various polysaccharides (e.g. capsular polysaccharides and lipopolysaccharides), N-linked glycoproteins, and flagellar O-linked glycoproteins of the *C. jejuni* define the collection of common glycans present on the surface of *C. jejuni*: galactose (Gal), N-acetylgalactosamine (GalNAc), and, to a lesser extent, N-acetylglucosamine (GlcNAc), mannose (Man), N-acetylneuraminic acid (Neu5Ac), and fucose (Fuc) (Corcoran and Moran, 2007; Turonova et al., 2016). Another structure present on the *C. jejuni* surface is the O-methyl phosphoramidate modification, which is important for cellular interactions and infection (Van Alphen et al., 2014). Interestingly, the presence of surface glycans seems to be strain-specific and affected by environmental factors, such as temperature (Day et al., 2009, 2013; Kilcoyne et al., 2014).

As previously shown by Klančnik et al (Klančnik et al., 2017a), fungal fruiting bodies represent a rich source of interesting antimicrobial and anti-adhesive substances. Of these, lectins and protease inhibitors (PIs) function as innate protein defense systems against amoebozoans, insects, and nematodes. After ingestion, lectins bind to specific glycans in the digestive tract and can cause cell disruption, whereas PIs inhibit the digestive proteases of fungivores (Sabotič et al., 2015). We hypothesize that fungal lectins may bind to specific glycans of *C. jejuni*, glycans on biotic surfaces, and intestinal epithelial cells, while PIs affect the enzymes secreted by *C. jejuni*, consequently affecting the attachment of *C. jejuni* to different surfaces. Therefore, the aim of this study was to evaluate fungal lectin- and PI-mediated modulation of *C. jejuni* adhesion to model surfaces and Caco-2 cells and *C. jejuni* invasion of Caco-2 cells.

## 2 Methods

### 2.1 Bacterial strain and growth conditions

*Campylobacter jejuni* NCTC 11168 (National Collection of Type Cultures) was used in this study. The strain was stored at −80°C in 20% glycerol (Kemika, Croatia) and 80% Mueller Hinton broth (Oxoid, UK). The strain was cultivated on Karmali agar (Oxoid, UK) with a selective supplement (SR0167E, Oxoid, UK) for 24 h at 42°C in a microaerobic atmosphere (85% N_2_, 10% CO_2_, 5% O_2_). Cultures were transferred to Mueller Hinton agar (Oxoid, UK) and incubated again for 24 h at 42°C in a microaerobic atmosphere. Bacterial concentration was determined spectrophotometrically (Perkin Elmer, USA) to 0.1 optical density (OD) at 600 nm. The suspension was further diluted by transferring 0.1 mL of the suspension to 9.9 mL of 2× Mueller Hinton broth to obtain an inoculum of approximately 1 × 10^5^ cells/mL. For the Caco-2 assay, a concentration of 1 × 10^8^ cells/mL (OD_600 =_ 0.2) was prepared in Dulbecco’s modified Eagle’s medium (DMEM).

### 2.2 Fungal lectins and PIs

Recombinant fungal lectins and PIs were prepared in the *Escherichia coli* expression system using different expression vectors. The following recombinant proteins were prepared as previously described: *Clitocybe nebularis* lectin [CNL, UniProt ID: B2ZRS9 (Pohleven et al., 2012)]; *Macrolepiota procera* lectin [MpL, Uniprot ID: F6KMV5 (Žurga et al., 2014)]; the macrocypins (Sabotič et al., 2009) macrocypin 1 (Mcp1, Uniprot ID: B9V973), macrocypin 3 (Mcp3, Uniprot ID: B9V979), and macrocypin 4 (Mcp4, Uniprot ID: B9V982); cocaprin 1 [CCP1, Uniprot ID: A8PCJ3 (Renko et al., 2022)]; and cospin [PIC, Uniprot ID: D0EWJ0 (Sabotič et al., 2012)].

The following lectins were produced as His-tagged recombinant proteins in *E. coli* BL21(DE3) using ZYM-5052 autoinduction medium for 4 h at 37°C and 20 h at 18°C (Studier, 2005): *Coprinopsis* galectin 2 (CGL2, UniProt ID: Q9P4R8 [Walser et al., 2004)], *Coprinopsis* galectin 3 (CGL3, UniProt ID: Q206Z5 [Wälti, 2008)], *Marasmius oreades* agglutinin [MOA, UniProt ID: Q8X123 (Kruger et al., 2002; Grahn et al., 2007)], *Agaricus bisporus* lectin [ABL, UniProt ID: Q00022 (Carrizo et al., 2005)], the *Sordaria macrospora* transcript associated with perithecial development [TAP1, UniProt ID: F7VWP8 (Nowrousian and Cebula, 2005)], *Aleuria aurantia* lectin [AAL, UniProt ID: P18891 (Wimmerova et al., 2003; Olausson et al., 2011)], *Coprinopsis cinerea* lectin 2 [CCL2, UniProt ID: B3GA02 (Schubert et al., 2012)], *C. cinerea* mucin-binding lectin 1 [CML1, UniProt ID: B3VS76 (Bleuler-Martinez et al., 2022)], and *Laccaria bicolor* tectonin 2 [Tec2, UniProt ID: B0CZL6 (Wohlschlager et al., 2014)].

Bacteria were harvested with centrifugation and sonicated in buffer A (30 mM Tris, pH 7.5, 400 mM NaCl) with 1 mg/mL lysozyme and cOmplete™ EDTA-free Protease Inhibitor Cocktail (Roche, Switzerland). Lectins were purified (**Supplementary Figure 1**) using a two-step purification protocol with His-tag affinity chromatography [HisTrap FF 5 mL column (Cytiva, USA) in buffer A with 10 mM imidazole for binding and 300 mM imidazole for elution] and size-exclusion chromatography [HiPrep 26/60 Sephacryl S-200 HR or HiPrep 26/60 Sephacryl S-100 HR column (Cytiva, USA) in buffer A]. They were then stored in phosphate-buffered saline (PBS; Oxoid, UK) in aliquots at −20°C until use. The plant lectin concanavalin A (ConA; L-1000, Vector Labs) was used as a control.

### 2.3 Preparation of model biotic surfaces on microtiter plates

Biotic surfaces were prepared with collagen type I solution from rat tail, fibronectin from human plasma, and mucin type II from porcine stomach (all from Sigma Aldrich, Germany). Stock solutions of these macromolecules were prepared according to the manufacturer’s instructions and were diluted with sterile PBS to obtain working solutions. For mucin type II, we chose a concentration of 1 mg/mL (Shi et al., 2000; Celebioglu et al., 2017). For collagen and fibronectin, we optimized the concentrations based on the largest number of adherent *C. jejuni* cells and chose 50 µg/mL and 10 µg/mL, respectively. To prepare the biotic surfaces, 200 µL of each solution at the working concentration was added to each well and incubated for 24 h at 4°C. Excess solution was carefully removed, and the wells were washed with sterile PBS immediately before experiments.

### 2.4 Testing the effects of lectins and PIs on *C. jejuni* adhesion to abiotic and biotic model surfaces

The effects of lectins on *C. jejuni* adhesion were tested on one abiotic and three biotic model surfaces. Nunc 96-well polystyrene microtiter plates (Thermo Fisher, USA) were used to test *C. jejuni* adhesion to abiotic surfaces and to prepare biotic model surfaces.

Before testing lectin-mediated modulation of *C. jejuni* adhesion, we tested whether lectins (at different concentrations) affect *C. jejuni* growth during 24 h incubation at 42°C (**Supplementary Figure 2**) by measuring OD_600_ at inoculation and at the end of cultivation (Varioskan LUX, Thermo Fisher, USA). The effects of 17 lectins (**Table 1**) on the adhesion of *C. jejuni* to abiotic and biotic model surfaces were tested at 250 µg/mL, except for MOA, which was used at 125 µg/mL due to its inhibitory effect on growth at 250 µg/mL. The stock solutions of lectins were diluted to 500 µg/mL with sterile PBS buffer and mixed with the *C. jejuni* inoculum in each well of the microtiter plate at a ratio of 1:1 to a final volume of 200 µL. The microtiter plate was shaken (600 rpm, 2 min) (Thermo Fisher, USA) and incubated for 24 h at 42°C in a microaerobic atmosphere. Then each well was washed three times, and adherent cells were removed by sonication followed by Koch’s dilution and drop plating, as described in (Klančnik et al., 2017b). After incubation for 24 h at 42°C in a microaerobic atmosphere, colony-forming units (CFU) on plates were counted. Experiments were conducted in three independent biological and technical replicates.

**Table 1:** Fungal proteins (lectins and protease inhibitors) used in this study with additional information regarding their origin, UniProt code, structure, specificity, and study reference.

Abbrev.	Full name and source species	UniProt code	Protein structure group and PDB	Type and specificity (glycan-binding or protease inhibition)	References
**ConA**	Concanavalin A (*Canavalia ensiformis)*	CONA_CANEN	beta-sandwich/ConA-like (1CVN), P02866	Lectin – Glc, Man, GlcNAc	
**CNL**	*Clitocybe nebularis* lectin	CNL_CLINE	beta-trefoil (3NBC), B2ZRS9	Lectin – GalNAcβ1-4GlcNAc	Pohleven et al., 2012
**MpL**	*Macrolepiota procera* lectin	F6KMV5_MACPC	beta-trefoil (4ION), F6KMV5, F6KMV5_MACPC	Lectin – Gal-β1-4-GlcNAc	Žurga et al., 2014
**CGL2**	*Coprinopsis* galectin 2 (*Coprinopsis cinerea)*	CGL2_COPCI	beta-sandwich/ConA-like (1UL9), Q9P4R8	Lectin – Gal-β1,4-Glc/GlcNAc/Fuc, Gal-β1,3-GalNAc	Walser et al., 2004
**CGL3**	*Coprinopsis* galectin 3 (*Coprinopsis cinerea)*	CGL3_COPCI	beta-sandwich/ConA-like (2R0F), Q206Z5	Lectin – GalNAcβ1-4GlcNAc	Wälti, 2008
**MOA**	*Marasmius oreades* agglutinin	Q8X123_9AGAR	beta-trefoil chimeric with a proteolytic domain (2IHO), Q8X123	Lectin – Gal-α1,3-Gal/GalNAc-β	Kruger et al., 2002; Grahn et al., 2007
**ABL**	*Agaricus bisporus* lectin	ABL_AGABI	beta-sandwich/cytolysin-like (1Y2T), Q00022	Lectin – Gal-β1,3-N-GalNAc and Gal-β1,3-N-GlcNAc	Carrizo et al., 2005
**TAP1**	*Sordaria macrospora* transcript associated with perithecial development	F7VWP8_SORMK	beta-sandwich, F7VWP8	Lectin – Gal-β1,3-GalNAc	Nowrousian & Cebula, 2005
**AAL**	*Aleuria aurantia* lectin	LECF_ALEAU	beta-propeller (1OFZ), P18891	Lectin – α1-2-, α1-3-, or α1-6-linked fucose	Wimmerova et al., 2003; Olausson et al., 2008
**CCL2**	*Coprinopsis cinerea* lectin 2	B3GA02_COPCI	beta-trefoil (2LIE), B3GA02	Lectin – Gal/GlcNAc-β1,4(Fuc-α1,3)	Schubert et al., 2012
**CML1**	*Coprinopsis* mucin-binding lectin 1 (*Coprinopsis cinerea)*	B3VS76_COPCI	beta-sandwich/agaromycete-like (6ZRW), B3VS76	Lectin – α1-2-, α1-3-, or α1-4-linked fucose	Bleuler-Martinez et al., 2022
**Tec2**	*Laccaria bicolor* tectonin 2	B0CZL6_LACBS	beta-propeller (5FSB), B0CZL6	Lectin – 3-O-Me-Man; 4-O-Me-Man*; 2-O-Me-Fuc	Wohlschlager et al., 2014
**CCP1**	Cocaprin 1 (*Coprinopsis cinerea)*	UniRef100_UPI002023BBEC	beta-trefoil (7ZNX)	Protease inhibitor – cysteine and aspartic proteases	Renko et al., 2022
**PIC**	Cospin (*Coprinopsis cinerea)*	D0EWJ0_COPCI	beta-trefoil (3N0K), D0EWJ0	Protease inhibitor – serine protease (trypsin)	Sabotič et al., 2012
**Mcp1**	Macrocypin 1 (*Macrolepiota procera)*	MCP1A_MACPC	beta-trefoil, B9V973	Protease inhibitor – cysteine proteases	Sabotič et al., 2009
**Mcp3**	Macrocypin 3 (*Macrolepiota procera)*	B9V979_MACPC	beta-trefoil, B9V979	Protease inhibitor – cysteine proteases	Sabotič et al., 2009
**Mcp4**	Macrocypin 4 (*Macrolepiota procera)*	B9V983_MACPC	beta-trefoil, B9V983	Protease inhibitor – cysteine proteases	Sabotič et al., 2009

### 2.5 Caco-2 cell cultivation

Caco-2 cells derived from human colon adenocarcinomas are a reliable model for the study of adhesion and invasion, thanks to their differentiation into cells that mimic intestinal epithelial cells with tight junctions, microvilli and specific enzymes and transporters (Arndt et al., 2011; Wong et al., 2018). These cells are known to facilitate studies on gut function and microbial interactions and represent a stable, reproducible and manageable alternative to primary or non-cancerous cell lines, where viability and complexity of culture often pose problems. Therefore, Caco-2 cells are invaluable for studying the pathogenesis of *C. jejuni* and shed light on the function of the intestinal barrier and the mechanisms of bacterial adhesion and invasion.

Caco-2 cell line (DSMZ no. ACC 169) was acquired from Leibniz Institute – DSMZ (Braunschweig, Germany) and was cultured in DMEM (Sigma-Aldrich, USA), supplemented with 5% (v/v) foetal bovine serum (Gibco, USA), 2 mM L-glutamine, 100 units/mL penicillin, and 100 µg/mL streptomycin (all from Merck, Germany) in tissue culture flasks at 37°C in a humidified 5% CO_2_ atmosphere. Caco-2 cells were seeded into 96-well tissue culture plates (Corning, USA) at 100 µL/well for the cytotoxicity assay (at 1 × 10^5^ cells/mL) and adhesion and invasion assay (at 7 × 10^5^ cells/mL) and grown as described above until confluence.

### 2.6 Cytotoxicity assay on Caco-2 cells

The cytotoxic effects of selected lectins (ConA, CGL2, CGL3, MOA, AAL, Tec2, and CCL2) were determined using the 3-(4,5-dimethylthiazol-2-yl)-2,5-diphenyltetrazolium bromide (MTT) colorimetric assay (Mosmann, 1983). After 48 h of incubation, Caco-2 cells were treated with lectins diluted in DMEM supplemented with 5% foetal bovine serum. For each lectin concentration (250–0.24 μg/mL), 100 µL/well was added to the plates in six replicates, with three repetitions. Afterwards, MTT solution (5 mg/mL) was prepared in PBS and added to each well. Microtiter plates were shaken for 5 min and then incubated for another 4 h at 37°C under 5% CO_2_. Then the MTT solution was carefully removed, and plates were left to dry overnight. After the addition of acid-isopropanol (0.04% HCl), the absorbance of the samples was measured using a microplate reader at 570 and 630 nm. Wells containing cells without lectins served as negative controls. In this way, we obtained non-toxic concentrations of lectins for Caco-2 cells, which were used for further studies: 250 µg/mL ConA, 60 µg/mL CGL2, 60 µg/mL CGL3, 0.24 µg/mL MOA, 250 µg/mL AAL, 10 µg/mL Tec2, and 250 µg/mL CCL2 (**Supplementary Table 1**).

### 2.7 Testing the effects of lectins on *C. jejuni* adhesion to and invasion of Caco-2 cells

To evaluate the preventive effectiveness of selected fungal lectins in reducing *C. jejuni* adhesion to and invasion of Caco-2 cells, we implemented three distinct incubation treatments. These treatments allowed sufficient time for the lectin to adhere to either *C. jejuni* cells (Treatment I) or Caco-2 cells (Treatment II), or simultaneous inoculation with *C. jejuni* cells into the Caco-2 cell culture (Treatment III).

#### 2.7.1 Treatment I: pre-incubation of lectins with *C. jejuni* before application to Caco-2 cells

Lectins were prepared in 1 mL of DMEM to each non-toxic concentration (defined above) to obtain working solutions. Bacterial inoculums in DMEM (1 mL) containing no antibiotics and with approximately 10^8^ CFU/mL of *C. jejuni* were suspended in the lectin solutions and incubated for 1 h at 42°C in a microaerobic atmosphere. This mixture was then added to confluent monolayers of Caco-2 cells and incubated for 2 h at 37°C in a humified atmosphere containing 5% CO_2_.

#### 2.7.2 Treatment II: pre-incubation of lectins with Caco-2 cells before adding *C. jejuni*

Working solutions of lectins were added to Caco-2 cell monolayers and incubated for 1 h at 37°C under 5% CO_2_. Then, bacterial inoculums were added to cell monolayers and incubated for 2 h at 37°C in a humified atmosphere containing 5% CO_2_ to allow bacterial adhesion.

#### 2.7.3 Treatment III: simultaneous inoculation of lectins and *C. jejuni*

Working solutions of lectins and bacterial inoculums were inoculated simultaneously to cell monolayers, and the plates were incubated for 2 h at 37°C in a humified atmosphere containing 5% CO_2_ to allow adhesion and invasion.

Controls without lectins, prepared by adding only bacterial inoculum, were kept under the same conditions. The effects of lectins on the adhesive and invasive ability of *C. jejuni* were observed in biological and technical triplicates. After washing twice with 200 μL of DMEM without antibiotics, DMEM containing 100 μg/mL gentamicin (Sigma Aldrich, Germany) was added to determine the number of invaded *C. jejuni*. After 1 h, the monolayers were lysed with 200 μL of 1 mL/L (v/v) Triton-X100 (Sigma Aldrich, Germany). The number of intracellular bacteria were determined for all three treatments with cultivability assays 24 h post-infection. A similar procedure but without gentamicin treatment (Šikić Pogačar et al., 2009) was performed to determine the total number of adherent and internalized bacteria. The difference between the numbers of total and intracellular bacteria was calculated to obtain the number of adherent *C. jejuni* cells.

### 2.8 Statistical analysis

The results were expressed as mean ± standard deviation of LOG_10_ (CFU/mL) (**Supplementary Tables 2, 4**). To calculate all relative differences, the mean logarithmic value of a treated sample was subtracted from the mean logarithmic value of an untreated sample. The absolute antilog value of the difference was expressed as a percentage with mean standard deviation (±%). Statistical analysis was performed with IBM SPSS Statistics 23 (Statsoft Inc., USA). A Shapiro-Wilk test of normality was performed to determine the distribution of the data. One-way ANOVA with Brown-Forsythe test was used, and a *p*-value of < 0.05 was considered statistically significant. Correlation analysis using Pearson’s test and graphical representation of all statistical analyses were performed using Graphpad prism 10.1 (GraphPad Software, USA).

## 3 Results

First, we tested whether the lectins have any effect on *C. jejuni* growth (**Supplementary Figure 1**). The lectins and PIs (at 250 µg/mL) did not affect growth, except MOA, which showed an inhibitory effect at 250 µg/mL and was thus used at 125 µg/mL (**Supplementary Figure 2**). Therefore, we assumed that lectins at the selected concentrations do not reduce *C. jejuni* growth but affect other bacterial characteristics, of which we tested adhesion to different surfaces and Caco-2 cell invasion.

### 3.1 Lectin and PI-mediated modulation of *C. jejuni* adhesion to abiotic and biotic model surfaces

**Figure 2:**
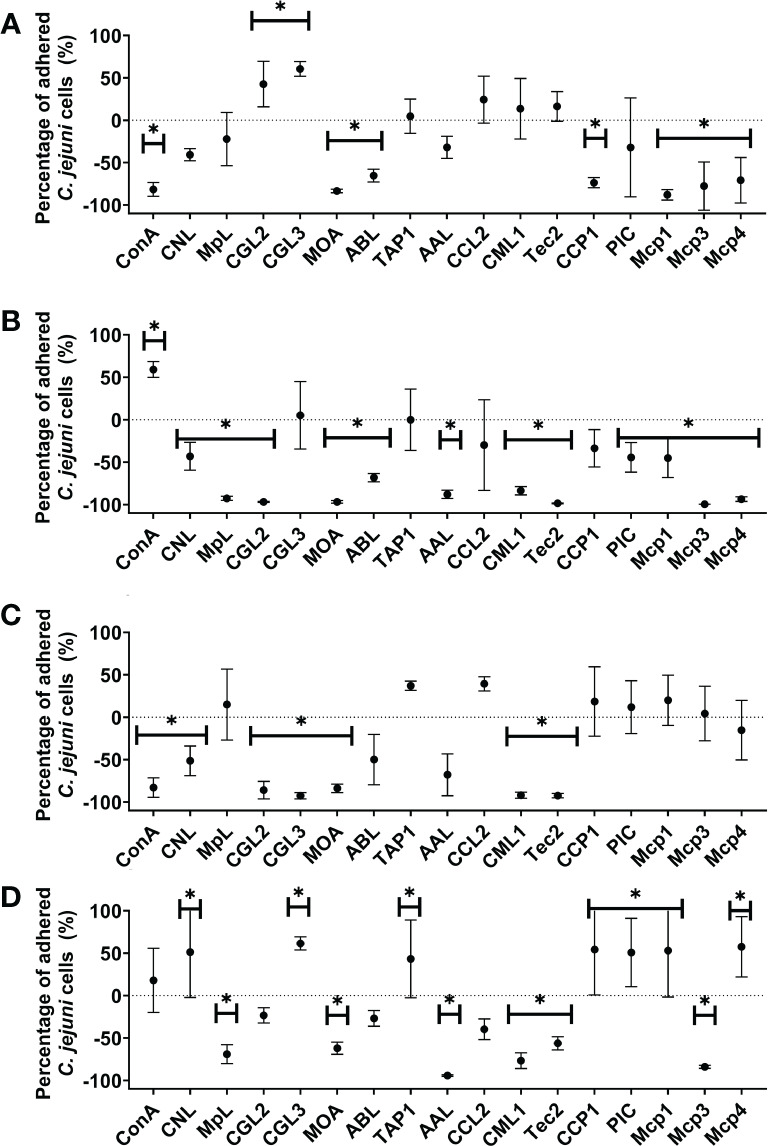
The effects of fungal lectins and protease inhibitors (PIs) on *Campylobacter jejuni* adhesion **(A–D)**. Relative differences between *C jejuni* adhered cells to **(A)** abiotic (polystyrene) and **(B–D)** biotic surfaces [mucin type II **(B)**, fibronectin **(C)**, and collagen type I **(D)**] after 24 h of co-incubation with or without lectins or PIs (% ± SD). Statistically significant changes are marked with *(*p-*value< 0.05). Experiments were conducted in three independent biological and technical replicates. Abbreviations are defined in **Table 1**.

The effects of 12 lectins (ConA as a control lectin and 11 lectins with known glycan-binding specificity) and 5 PIs on *C. jejuni* adhesion to abiotic and biotic (mucin, fibronectin, and collagen) model surfaces were tested (**Figure 2**, **Supplementary Tables 2, 3**). The effects were observed as significant decreases or increases in the numbers of adhered *C. jejuni* cells on the selected surfaces. CCL2 was the only lectin that did not significantly affect *C. jejuni* adhesion to abiotic or biotic model surfaces. Of the 17 lectins and PIs, 12 significantly decreased *C. jejuni* adhesion to mucin type II, 7 significantly decreased *C. jejuni* adhesion to polystyrene and fibronectin, and 6 significantly decreased *C. jejuni* adhesion to collagen type II. MOA was the only lectin that effectively decreased *C. jejuni* adhesion to all selected surfaces. CML1 and Tec2 significantly decreased *C. jejuni* adhesion to all biotic model surfaces but not polystyrene. CCP1 significantly decreased *C. jejuni* adhesion only to polystyrene.

Conversely, certain lectins and PIs also increased *C. jejuni* adhesion to collagen type II (7), polystyrene, (2), and mucin type (1). None of the lectins increased *C. jejuni* adhesion to fibronectin. Interestingly, TAP1 significantly increased *C. jejuni* adhesion to collagen but did not affect adhesion to any other surface (**Supplementary Table 3**).

Correlation analysis between biotic surfaces using Pearson’s method was also performed (**Supplementary Figure 3**). The results show a strong correlation between the effects of selected lectins and PIs on the modulation of *C. jejuni* adhesion to collagen type I and mucin type II (Pearson correlation coefficient value of 0.59 and *P*-value of 0.01).

### 3.2 *C. jejuni* adhesion to and invasion of Caco-2 cells

**Figure 3:**
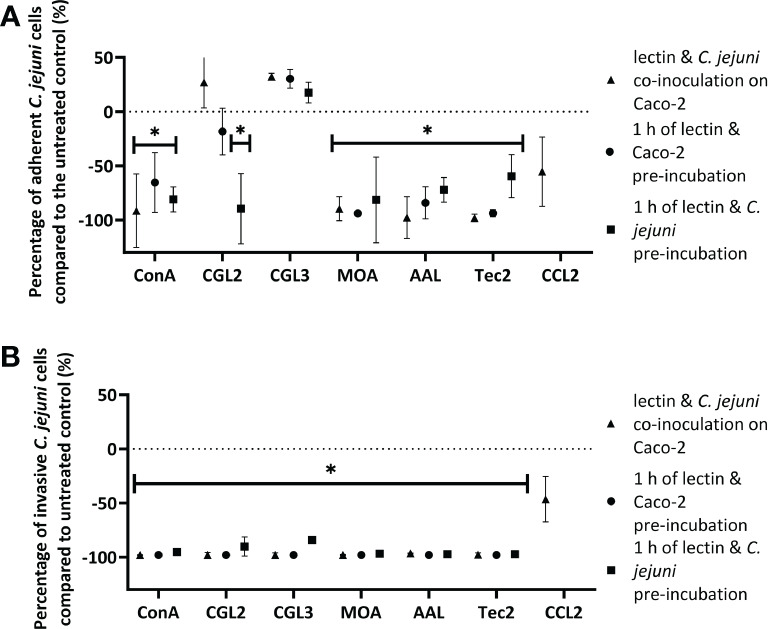
The effects off fungal lectins on *Campylobacter jejuni* adhesion to and invasion of Caco-2 cells. Values are given as relative changes in the numbers of adherent **(A)** or invasive **(B)** *C jejuni* cells relative to the untreated control (% ± SD). Statistically significant changes are marked with *(*p-*value< 0.05). Three conditions were tested: 2 h of co-incubation of lectins, *C jejuni*, and Caco-2 cells (triangles), 1 h of pre-incubation of lectins with Caco-2 cells (circles), and 1 h of pre-incubation of lectins with *C jejuni* (squares) before the final 2 h co-incubation of *C jejuni* with Caco-2 cells. Due to the lack of effect in adherent or invaded *C jejuni* cells, the lectin CCL2 was only tested using the method of simultaneous co-inoculation of lectin and *C jejuni* on Caco-2 cells. Experiments were conducted in three independent biological and technical replicates. Abbreviations are defined in **Table 1**.

**Figure 4:**
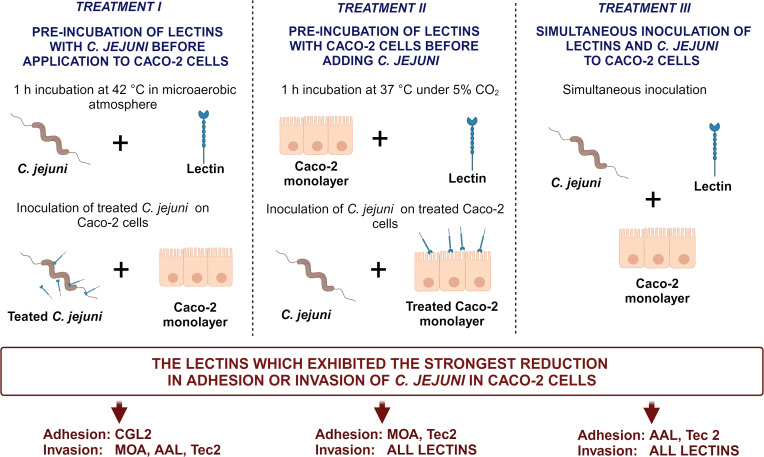
Schematic overview of three distinct (pre-)incubation procedures in the Caco-2 adhesion and invasion assay. The lectins yielding the most pronounced reduction for each treatment are listed at the bottom of the figure. The image was created using BioRender.com. Abbreviations are defined in **Table 1**.

To further evaluate the anti-adhesive potential of fungal lectins, an adhesion and invasion assay with *C. jejuni* and human intestinal epithelial Caco-2 cells was employed (**Figure 3**, **Supplementary Tables 4, 5**). For this purpose, the lectins that decreased *C. jejuni* adhesion to different model surfaces by more than 90% (**Supplementary Table 3**) i.e. CGL2, CGL3, MOA, AAL, and Tec2, as well as control lectin ConA were selected. For the negative control, CCL2 was chosen as it did not affect *C. jejuni* adhesion to the model surfaces (**Supplementary Table 3**). Non-toxic lectin concentrations, which were determined by evaluating the effects of lectins on cell proliferation, were used (**Supplementary Table 1**). In addition, different pre-incubation combinations of lectins, *C. jejuni*, and Caco-2 cells were used (**Figure 4**). The purpose of these pre-incubations was to determine whether the binding of lectins to either bacterial or Caco-2 cells plays a more important role in the observed decrease in the number of adherent or invasive cells during the 2 h co-incubation (**Figure 3**).

Lectin-mediated modulation of *C. jejuni* adhesion to biotic surfaces and Caco-2 cells differed between different lectins (**Supplementary Tables 3, 5**). Furthermore, the type of pre-incubation also affected adhesion to Caco-2 cells (**Figures 3**, **4**). ConA, MOA, AAL, and Tec2 decreased adhesion when incubated simultaneously with *C. jejuni* and when pre-incubated with Caco-2 cells for 1 h. Conversely, CGL2 and CGL3 did not affect *C. jejuni* adhesion when co-inoculated with *C. jejuni*, whereas pre-incubation of CGL2 with *C. jejuni* decreased adhesion. Pre-incubation of CGL2 with both *C. jejuni* and Caco-2 cells decreased bacterial adhesion to Caco-2 cells and even more notably decreased *C. jejuni* invasion of Caco-2 cells (**Figure 3**, **Supplementary Table 5**). Simultaneous incubation decreased invasion below the detection limit of our method (< 2 log CFU/mL) for ConA, CGL2, CGL3, and MOA and by almost a factor of 2 log values for AAL and Tec2. After lectins and Caco-2 cells were pre-incubated for 1 h, no invasion was detected for any of the lectins (< 2 log CFU/mL). Pre-incubation of lectins and *C. jejuni* for 1 h was least effective compared to other pre-incubation combinations, as invasion was observed for all treated Caco-2 cells (**Figure 3**, **Supplementary Tables 4, 5**). Nevertheless, the number of invaded cells was still decreased by a factor of 1 log.

## 4 Discussion

Understanding surface adhesion, which is an early stage of biofilm formation, is crucial for developing reliable and efficient prevention strategies to control foodborne pathogens. Therefore, we tested whether fungal (glycan-binding) lectins and PIs can reduce the presence of *C. jejuni* cells on different surfaces. Our results show that different lectins can increase or decrease adhesion with varying efficacy depending on the surface. We can thus assume that *C. jejuni* adapts and utilizes different adhesion processes depending on the surface it comes into contact with.

In this study, *C. jejuni* adhesion to an abiotic surface (polystyrene) was decreased by the lectins MOA, ConA, and ABL and the PIs CCP1, Mcp1, Mcp3, and Mcp4. Polystyrene is a commonly used material in the food industry and is particularly well colonized by microorganisms. Hydrophobic interactions are the main driving force for the initial attachment of *C. jejuni* cells to such surfaces (Katsikogianni et al., 2004). As suspected, lectins and PIs had limited effects (less than 90% decrease in adhesion) on interactions between *C. jejuni* and the abiotic surface, as polystyrene has no specific glycan targets.

Many of the lectins that were most potent in decreasing adhesion to mucin, e.g. MOA, AAL, ABL, CML1, and Tec2, have a glycan-binding target that is present on mucins and the surface of *C. jejuni* (Kruger et al., 2002; Wimmerova et al., 2003; Carrizo et al., 2005; Corcoran and Moran, 2007; Grahn et al., 2007; Olausson et al., 2008; Wohlschlager et al., 2014; Turonova et al., 2016; Bleuler-Martinez et al., 2022). The main glycans of mucin type II are GalNAc, GlcNAc, Fuc, Gal, and Neu5Ac (Schömig et al., 2016). Conversely, the core oligosaccharides of *C. jejuni* lipopolysaccharides consist of 3-deoxy-α-D-manno-oct-2-ulopyranosonic acid, L-glycero-D-manno-heptose, glucose (Glc), Gal, Neu5Ac, and GalNAc (Karlyshev et al., 2005). As the first line of gastrointestinal tract defense, mucins provide a convenient target for gastrointestinal pathogens to adhere to and subsequently invade the cells of the intestinal epithelium (McGuckin et al., 2011). As an essential chemoattractant for *C. jejuni*, mucins are also critically involved in *C. jejuni* colonization of the underlying epithelium, which requires increased flagellar gene expression and motility (Reid et al., 2008) Our results show that many lectins decrease adhesion, suggesting that they may prevent *C. jejuni* invasion of host cells by preventing the initial attachment and transition of *C. jejuni* into its pathogenic phenotype (Hong et al., 2014).

*Campylobacter jejuni* had the strongest affinity for fibronectin compared with the other surfaces tested. Three lectins (CGL3, CML1, and Tec2) decreased adhesion by more than 90%. Fibronectin is known to be a target for numerous bacterial proteins (from both Gram-positive and Gram-negative bacteria), which generally function as bacterial adhesins, i.e. fibronectin-binding proteins (Henderson et al., 2011). These proteins are critical for the first step of host cell invasion (Talukdar et al., 2020; Konkel et al., 2020). Fibronectin has fewer glycan-binding sites than the other biotic surfaces used in this study and is mainly composed of Man-, Neu5Ac-, Fuc-, GlcNAc-, and Gal-binding sites (Hsiao et al., 2017). However, the *C. jejuni* adhesins CadF and FlpA target the protein part of fibronectin (Konkel et al., 2020). This may explain the lower number of lectins that can effectively decrease adhesion to fibronectin compared to other biotic surfaces (**Supplementary Table 4**), highlighting the importance of finding an effective approach for inhibiting *C. jejuni* adhesion to fibronectin.

Surprisingly, many lectins and PIs (e.g. CGL3 and Mcp1) increased the number of *C. jejuni* cells that adhered to collagen but decreased the number of *C. jejuni* cells that adhered to other biotic surfaces. Collagen is the target of surface-anchored adhesins and other virulence factors (produced by both Gram-positive and Gram-negative bacteria) that mediate adhesion to host cells and tissues and enable colonization, invasion, and biofilm formation (Singh et al., 2012; Arora et al., 2021). Glycosylation of collagen consists of Gal and Glc-Gal units forming the disaccharide Glc(α1-2)-Gal(β1-O) (Hennet, 2019). This bottleneck in glycan diversity may explain why certain lectins did not affect adhesion. Interestingly, the lectins that most increased *C. jejuni* adhesion (CGL3, TAP1, and CNL) have similar targets (GalNAcβ1-4GlcNAc and Gal-β1,3-GalNAc). Conversely, the lectin MpL has the specific target Gal-β1-4-GlcNAc and decreased adhesion to collagen, highlighting the complex mechanism underlying *C. jejuni* adhesion.

Although we do not know whether the lectins used in this study bind to glycans on *C. jejuni* or the tested biotic surfaces, their effects on adhesion were surface-dependent. MOA was the most effective and only lectin to decrease adhesion to all surfaces tested (abiotic and biotic). The specific glycan target of MOA is Gal-α1,3-Gal/GalNAc, and Gal subunits are found on all the biotic surfaces used in this study and the surface of *C. jejuni*. Thus, the possibility of a synergistic effect should be considered. Day et al (Day et al., 2009). showed that *C. jejuni* 11168 can bind to Gal, Fuc, Neu5Ac, Man, glucosamine, and glycosaminoglycans. Therefore, we can argue that MOA might outcompete *C. jejuni* for binding sites on surfaces. Furthermore, CML1 and Tec2 decreased *C. jejuni* adhesion to all biotic surfaces. CML1 decreased adhesion to fibronectin by more than 90%, whereas Tec2 decreased adhesion to fibronectin and mucin type II by more than 90%.

In addition, we performed a correlation analysis using Pearson’s method (**Supplementary Figure 3**), which shows a strong correlation between the effects of lectins on *C. jejuni* adhesion to collagen type I and mucin type II. This substantiates our results because both these biotic surfaces contain the same glycans consisting of Gal and Glc, common targets for the tested lectins.

For the Caco-2 assay, lectins (ConA, CGL2, CGL3, MOA, AAL, Tec2 and CCL2) were used in three different (pre-)incubation procedures, aiming to evaluate their effectiveness in inhibiting bacterial adhesion and invasion. These processes represent critical initial stages in *C. jejuni* infection and subsequent disease progression (**Figure 4**). CCL2 was selected as a control because it did not affect *C. jejuni* adhesion to the biotic surfaces studied. As expected, it did not significantly decrease *C. jejuni* adhesion or Caco-2 cell invasion, underscoring the validity of our previous tests on biotic surfaces. Furthermore, AAL, ConA, and MOA have previously been shown to bind to *C. jejuni* (Turonova et al., 2016), and Day et al (Day et al., 2009). showed that ConA can decrease adhesion of *C. jejuni* NCTC 11168 at 37°C, which is consistent with our results. ConA binds to Man and GlcNAc, which are found on the surface of Caco-2 cells, and to Glc, which is found on the surface of *C. jejuni*. Thus, the results differed depending on the pre-incubation assay, with the greatest decrease in adhesion and invasion in Caco-2 cells obtained when the selected lectin and *C. jejuni* were added simultaneously to Caco-2 cells (**Figure 3**).

CGL2 and CGL3 are similar lectins that bind to beta-galactosides. Interestingly, they did not decrease *C. jejuni* adhesion except after 1 h of pre-incubation of CGL2 and *C. jejuni*. Nevertheless, both lectins lowered *C. jejuni* invasion after simultaneous inoculation and 1 h pre-incubation of each lectin and Caco-2 cells compared with our method’s limit of detection. By contrast, MOA, which was used at a lower concentration than the other lectins, targets Gal or GalNAc disaccharides in α-configuration, i.e. glycans found on biotic surfaces such as Caco-2 and *C. jejuni* cells. It decreased adhesion and invasion in all combinations, confirming the results of our previous tests on biotic surfaces. In addition, AAL and Tec2 bind to Fuc and methylated Man/Fuc, respectively, which are found on the surface of Caco-2 cells (Wong et al., 2018), and had a similar effect as MOA.

As shown by Louwen et al (Louwen et al., 2008), *C. jejuni* adhesion and invasion are differentially regulated. Adhesion depends more on proteins such as fibronectin-binding proteins (Henderson et al., 2011), whereas invasion is more influenced by the glycan composition of the outer membrane of *C. jejuni*; in particular, sialylation plays an important role in epithelial cell invasion. The glycan composition of Caco-2 cells varies and changes during differentiation and maturation (Wong et al., 2018). This may explain why different studies, e.g. Arndt et al (Arndt et al., 2011). and Tao et al (Tao et al., 2008), showed different binding affinities of lectins to Caco-2 cells, indicating the importance of experimental conditions when comparing results. The application of lectins holds promising potential across various domains, including food safety protocols, therapeutic interventions, probiotic therapy development, and biomaterial functionalization. These endeavors collectively aim to address the growing challenge of antibiotic resistance (Konozy et al., 2022).

## 5 Conclusion

An anti-adhesion approach proves to be a viable strategy in mitigating the persistence of foodborne pathogens in food-processing environments and their ability to invade hosts and cause disease. The inhibitory effects of lectins and PIs on *C. jejuni* adhesion differs between polystyrene, biotic surfaces, and Caco-2 cells. Our results emphasize that when conducting anti-adhesion tests for screening purposes, it is essential to use specific biotic surfaces rather than polystyrene. This is due to the involvement of different adhesion mechanisms, leading to potentially significant variations in results across different surfaces. Additionally, our results were influenced by different pre-incubation approaches, emphasizing the crucial role of methodological approaches when assessing lectins and PIs. Notably, the fungal lectins MOA and Tec2 significantly decreased *C. jejuni* adhesion to all surfaces and invasion of Caco-2 cells, even at lower concentrations (0.24 µg/mL for MOA and 10 µg/mL for Tec2). This highlights their promising potential as an alternative preventive method to combat the global spread of *C. jejuni* or a promising adjuvant to already existing curative treatments.

## Data availability statement

The original contributions presented in the study are included in the article/**Supplementary Material**. Further inquiries can be directed to the corresponding author.

## Ethics statement

Ethical approval was not required for the studies on humans in accordance with the local legislation and institutional requirements because only commercially available established cell lines were used.

## Author contributions

BJ: Data curation, Formal analysis, Investigation, Methodology, Writing – original draft. MŠP: Formal analysis, Investigation, Methodology, Writing – review & editing. MS: Conceptualization, Data curation, Formal analysis, Investigation, Writing – review & editing. TT: Formal analysis, Investigation, Methodology, Writing – review & editing. KK: Formal analysis, Investigation, Methodology, Writing – review & editing. DT: Funding acquisition, Project administration, Writing – review & editing. TL: Funding acquisition, Investigation, Project administration, Writing – review & editing. JS: Conceptualization, Formal analysis, Funding acquisition, Investigation, Methodology, Project administration, Validation, Visualization, Writing – review & editing. AK: Conceptualization, Data curation, Funding acquisition, Investigation, Project administration, Resources, Supervision, Visualization, Writing – review & editing.
