# Supplementary material for: Modulation of Campylobacter jejuni adhesion to biotic model surfaces by fungal lectins and protease inhibitors
Source: Front Cell Infect Microbiol. 2024 Apr 22;14:1391758. doi: 10.3389/fcimb.2024.1391758 (PMC11075492; doi:10.3389/fcimb.2024.1391758)
Supplement: Supplementary file 1 [file DataSheet_1.docx]

Supplementary Material

**Supplementary table 1** Cytotoxicity assay to determine non-toxic concentrations of lectins for Caco-2 cells. The first non-toxic concentrations detected are displayed in bold and expressed as relative viability of treated Caco-2 cells compared to untreated Caco-2 cells (% ± SD; six replicates and three repetitions each). The lectin MOA was not tested (N.T.) at a concentration of 250 µg/mL due to its growth inhibitory effect on *C. jejuni* at this concentration. Abbreviations are defined in Table 1

| Lectin | Lectin concentration (µg/mL) | | | | | | | |
| --- | --- | --- | --- | --- | --- | --- | --- | --- |
|  | 250 | | 60 | | 10 | | 0.24 | |
|  | Viability (%) | ± SD | Viability (%) | ± SD | Viability (%) | ± SD | Viability (%) | ± SD |
| ConA | **96.2** | **5.9** | / | / | / | / | / | / |
| CGL2 | 89.7 | 5.1 | **96.4** | **4.7** | / | / | / | / |
| CGL3 | 91.4 | 10.5 | **98.3** | **4.5** | / | / | / | / |
| MOA | N.T. | / | 26.6 | 3.9 | 47.1 | 1.2 | **96.3** | **6.1** |
| AAL | 100.5 | 4.6 | / | / | / | / | / | / |
| Tec2 | 72.4 | 5.2 | 87.9 | 1.7 | **94.7** | **3.5** | / | / |
| CCL2 | 94.6 | 4.4 | / | / | / | / | / | / |

**Supplementary table 2** Logarithmic values of the mean number of colony-forming units/mL (LOG_10_ (CFU/mL)) of adherent *Campylobacter jejuni* cells on abiotic (polystyrene) and biotic surfaces (mucin type II, fibronectin, and collagen type I). Significant (* *P* ≤ 0.05; ** *P* ≤ 0.01; *** *P* ≤ 0.001) decreases (marked in blue) and increases (marked in red) in cell adherence after 24 h are bolded. Abbreviations are defined in Table 1

| Surface | Polystyrene | | | Mucin type II | | | Fibronectin | | | Collagen type I | | |
| --- | --- | --- | --- | --- | --- | --- | --- | --- | --- | --- | --- | --- |
| Lectin | LOG_10_ (CFU/mL) | SD | *P* | LOG_10_ (CFU/mL) | SD | *P* | LOG_10_ (CFU/mL) | SD | *P* | LOG_10_ (CFU/mL) | SD | *P* |
| Control | 6.256 | ±0.035 |  | 6.337 | ±0.034 |  | 6.499 | ±0.021 |  | 5.751 | ±0.047 |  |
| ConA | **5.522** | ±0.093 | *** | **6.726** | ±0.063 | * | **5.731** | ±0.095 | ** | 5.837 | ±0.072 |  |
| CNL | 6.029 | ±0.130 |  | **6.092** | ±0.028 | ** | **6.186** | ±0.046 | ** | **6.063** | ±0.037 | ** |
| MpL | 6.147 | ±0.098 |  | **5.208** | ±0.035 | *** | 6.569 | ±0.042 |  | **5.242** | ±0.044 | *** |
| CCP1 | **5.675** | ±0.107 | ** | 6.159 | ±0.031 |  | 6.588 | ±0.036 |  | **6.089** | ±0.044 |  |
| PIC | 6.088 | ±0.126 |  | **6.082** | ±0.029 | ** | 6.554 | ±0.022 |  | **6.059** | ±0.044 | * |
| Mcp1 | **5.334** | ±0.055 | *** | **6.076** | ±0.037 | ** | 6.596 | ±0.024 |  | **6.079** | ±0.047 | ** |
| Mcp3 | **5.605** | ±0.066 | *** | **3.989** | ±0.041 | *** | 6.518 | ±0.020 |  | **4.959** | ±0.034 | *** |
| Mcp4 | **5.721** | ±0.116 | * | **5.153** | ±0.041 | *** | 6.427 | ±0.062 |  | **6.123** | ±0.032 | *** |
| CGL2 | **6.497** | ±0.026 | *** | **4.845** | ±0.019 | *** | **5.647** | ±0.104 | ** | 5.636 | ±0.033 |  |
| CGL3 | **6.659** | ±0.066 | ** | 6.360 | ±0.051 |  | **5.376** | ±0.065 | *** | **6.166** | ±0.034 | *** |
| MOA | **5.476** | ±0.050 | *** | **4.838** | ±0.108 | *** | **5.709** | ±0.087 | ** | **5.331** | ±0.031 | *** |
| AAL | 6.089 | ±0.057 |  | **5.420** | ±0.085 | *** | 6.007 | ±0.145 |  | **4.514** | ±0.041 | *** |
| ABL | **5.796** | ±0.069 | ** | **5.840** | ±0.046 | *** | 6.199 | ±0.063 |  | 5.615 | ±0.049 |  |
| TAP1 | 6.277 | ±0.023 |  | 6.337 | ±0.036 |  | 6.700 | ±0.044 |  | **5.997** | ±0.027 | ** |
| CCL2 | 6.377 | ±0.048 |  | 6.183 | ±0.101 |  | 6.716 | ±0.113 |  | 5.531 | ±0.063 |  |
| CML1 | 6.319 | ±0.054 |  | **5.549** | ±0.034 | *** | **5.408** | ±0.058 | *** | **5.119** | ±0.064 | ** |
| Tec2 | 6.179 | ±0.077 |  | **4.514** | ±0.029 | *** | **5.378** | ±0.062 | *** | **5.393** | ±0.029 | *** |

**Supplementary table 3** The effects of fungal lectins and protease inhibitors on *Campylobacter jejuni* adhesion to model abiotic and biotic surfaces. Relative differences between *C. jejuni* adhered cells co-incubated (24 h) with or without lectins (% ± SD). Significant (*P* ≤ 0.05) decreases and increases in the number of adherent cells on different surfaces after 24 h are bolded. Experiments were conducted in three independent biological and technical replicates. Abbreviations are defined in Table 1

| Lectin/PI | Change in the percentage of adherent *C. jejuni* cells compared to the control | | | | | | | |
| --- | --- | --- | --- | --- | --- | --- | --- | --- |
|  | Polystyrene | SD (%) | mucin type II | SD (%) | fibronectin | SD (%) | collagen type II | SD (%) |
| ConA | **−81.6%** | 8.1 | **59.2%** | 9.3 | **−82.9%** | 11.4 | 18.0% | 37.8 |
| CNL | −40.7% | 7.1 | **−43.1%** | 16.4 | **−51.4%** | 17.4 | **51.3%** | 53.5 |
| MpL | −22.2% | 31.3 | **−92.6%** | 2.5 | 14.9% | 41.8 | **−69.0%** | 11.1 |
| CGL2 | **42.6%** | 26.8 | **−96.8%** | 0.5 | **−85.9%** | 10.3 | −23.3% | 9.0 |
| CGL3 | **60.5%** | 8.7 | 5.2% | 39.8 | **−92.5%** | 3.7 | **61.5%** | 7.7 |
| MOA | **−83.4%** | 2.1 | **−96.8%** | 1.5 | **−83.8%** | 5.0 | **−62.0%** | 7.2 |
| ABL | **−65.3%** | 7.5 | **−68.2%** | 4.9 | −49.9% | 29.7 | −26.9% | 9.2 |
| TAP1 | 4.7% | 20.3 | 0.0% | 36.2 | 37.1% | 5.4 | **43.3%** | 45.8 |
| AAL | −31.9% | 13.0 | **−87.9%** | 4.9 | −67.8% | 24.7 | **−94.2%** | 0.9 |
| CCL2 | 24.3% | 27.6 | -29.9% | 53.4 | 39.3% | 8.4 | −39.7% | 12.2 |
| CML1 | 13.5% | 35.7 | **−83.7%** | 4.9 | **−91.9%** | 3.5 | **−76.7%** | 9.2 |
| Tec2 | 16.3% | 17.5 | **−98.5%** | 0.5 | **−92.4%** | 2.5 | **−56.2%** | 7.7 |
| CCP1 | **−73.7%** | 5.9 | -33.6% | 22.0 | 18.5% | 40.9 | **54.2%** | 53.5 |
| PIC | −32.1% | 58.3 | **−44.4%** | 17.4 | 11.9% | 31.1 | **50.8%** | 40.3 |
| Mcp1 | **−88.0%** | 6.2 | **−45.2%** | 22.9 | 20.0% | 29.6 | **53.0%** | 54.7 |
| Mcp3 | **−77.7%** | 28.4 | **−99.6%** | 0.1 | 4.3% | 32.1 | **−83.9%** | 1.8 |
| Mcp4 | **−70.8%** | 26.8 | **−93.5%** | 2.7 | −15.3% | 35.1 | **57.5%** | 35.5 |

**Supplementary table 4** Logarithmic values of the mean number of colony-forming units/mL (LOG_10_ (CFU/mL)) of adherent or invaded *Campylobacter jejuni* cells quantified in the Caco-2 assay after the 2 h co-incubation and 1 h pre-incubation of lectins with Caco-2 cells or *C. jejuni* before the 2 h co-incubation. Significant (*P* ≤ 0.05) decreases (marked in blue) and increases (marked in red) in cell adherence or invasiveness after 2 h are bolded. Abbreviations are defined in Table 1; ND, not determined

| Lectin | Change in the percentage of adherent/invasive *C. jejuni* cells | | | | | | | | | | | | | |
| --- | --- | --- | --- | --- | --- | --- | --- | --- | --- | --- | --- | --- | --- | --- |
|  | Adhesion to Caco-2 cells (±LOG_10_ (CFU/mL)) | | | | | | | Invasion of Caco-2 cells (±LOG_10_ (CFU/mL)) | | | | | | |
|  | lectin & *C. jejuni* co-inoculation of Caco-2 | SD | 1 h of lectin & Caco-2 pre-incubation | SD | 1 h of lectin & *C. jejuni* pre-incubation | SD | lectin & *C. jejuni* co-inoculation of CaCo-2 | | SD | 1 h of lectin & Caco-2 pre-incubation | SD | 1 h of lectin & *C. jejuni* pre-incubation | SD |  |
| control | 7.431 | 0.407 | 6.972 | 0.046 | 7.121 | 0.172 | 3.710 | | 0.105 | 3.574 | 0.069 | 3.580 | 0.158 |  |
| ConA | **6.362** | 0.357 | **6.511** | 0.235 | **6.398** | 0.211 | **< 1.996** | | / | **< 1.996** | / | **2.255** | 0.120 |  |
| CGL2 | 7.568 | 0.094 | 6.884 | 0.087 | **6.137** | 0.342 | **2.362** | | 0.232 | **< 1.996** | / | **2.568** | 0.081 |  |
| CGL3 | 7.600 | 0.365 | 7.128 | 0.334 | 7.204 | 0.230 | **< 1.996** | | / | **< 1.996** | / | **2.778** | 0.358 |  |
| MOA | **6.447** | 0.254 | **5.753** | 0.143 | **6.387** | 0.559 | **1.998** | | 0.003 | **< 1.996** | / | **2.079** | 0.120 |  |
| AAL | **5.794** | 0.119 | **6.170** | 0.209 | **6.564** | 0.140 | **2.243** | | 0.236 | **< 1.996** | / | **2.000** | 0.040 |  |
| Tec2 | **5.699** | 0.067 | **5.766** | 0.194 | **6.727** | 0.135 | **2.079** | | 0.224 | **< 1.996** | / | **2.000** | 0.040 |  |
| CCL2 | 6.938 | 0.037 | ND | / | ND | / | 3.361 | | 0.308 | ND | / | ND | / |  |

**Supplementary table 5** The effects of fungal lectins on *Campylobacter jejuni* adhesion and invasion in the Caco-2 assay. Relative differences in the numbers of adherent or invasive cells after the 2 h co-incubation and 1 h pre-incubation of lectins with Caco-2 cells or *C. jejuni* before the 2 h co-incubation compared to those in the absence of lectins. Significant (*P* ≤ 0.05) decreases and increases in bacterial adhesion and invasion after 2 h are bolded. Experiments were conducted in three independent biological and technical replicates. Abbreviations are defined in Table 1; ND, not determined

| Lectin | Change in the percentage of adherent/invasive *C. jejuni* cells | | | | | | | | | | | | | |
| --- | --- | --- | --- | --- | --- | --- | --- | --- | --- | --- | --- | --- | --- | --- |
|  | Adhesion to Caco-2 cells | | | | | | | Invasion of Caco-2 cells | | | | | | |
|  | lectin & *C. jejuni* co-inoculation of Caco-2 | SD (%) | 1 h of lectin & Caco-2 pre-incubation | SD (%) | 1 h of lectin & *C. jejuni* pre-incubation | SD (%) | lectin & *C. jejuni* co-inoculation of CaCo-2 | | SD (%) | 1 h of lectin & Caco-2 pre-incubation | SD (%) | 1 h of lectin & *C. jejuni* pre-incubation | SD (%) |  |
| ConA | **−91.5%** | ±34.0 | **−65.4%** | ±27.6 | **−81.1%** | ±11.5 | **< −98.1%** | | ±0.4 | **< −98.1%** | ±0.6 | **−95.3%** | ±2.9 |  |
| CGL2 | 27.0% | ±23.6 | −18.4% | ±21.6 | **−89.6%** | ±32.4 | **< −98.1%** | | ±2.2 | **< −98.1%** | ±0.6 | **−90.3%** | ±8.8 |  |
| CGL3 | 32.2% | ±3.1 | 30.3% | ±8.7 | 17.5% | ±9.6 | **< −98.1%** | | ±1.9 | **< −98.1%** | ±0.6 | **−84.2%** | ±0.6 |  |
| MOA | **−89.6%** | ±11.0 | **−94.0%** | ±2.3 | **−81.5%** | ±39.5 | **< −98.1%** | | ±0.4 | **< −98.1%** | ±0.6 | **−96.8%** | ±1.3 |  |
| AAL | **−97.7%** | ±19.1 | **−84.2%** | ±14.8 | **−72.2%** | ±11.4 | **−96.6%** | | ±0.4 | **< −98.1%** | ±0.6 | **−97.4%** | ±2.7 |  |
| Tec2 | **−98.2%** | ±3.5 | **−93.8%** | ±3.2 | **−59.6%** | ±19.8 | **−****97.7%** | | ±1.6 | **< −98.1%** | ±0.6 | **−97.4%** | ±0.4 |  |
| CCL2 | −55.4% | ±31.9 | ND |  | ND |  | −46.48% | | ±20.9 | ND |  | ND |  |  |

<
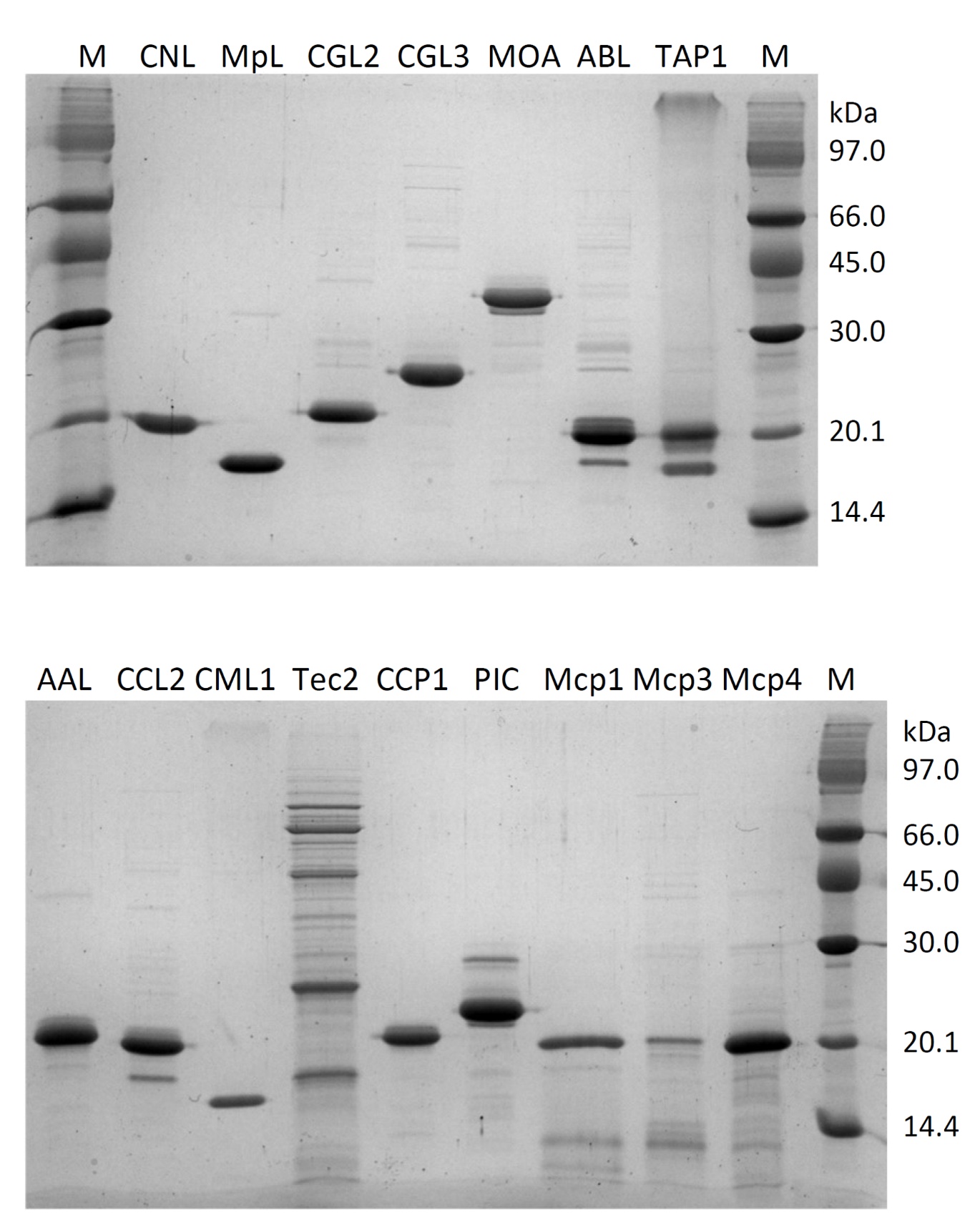
**Supplementary figure 1** Purified recombinant fungal lectins and protease inhibitors (2.5 µg/mL) were analyzed by SDS-PAGE (15%) and visualized by Coomassie staining. Abbreviations are defined in Table 1; M: molecular mass marker


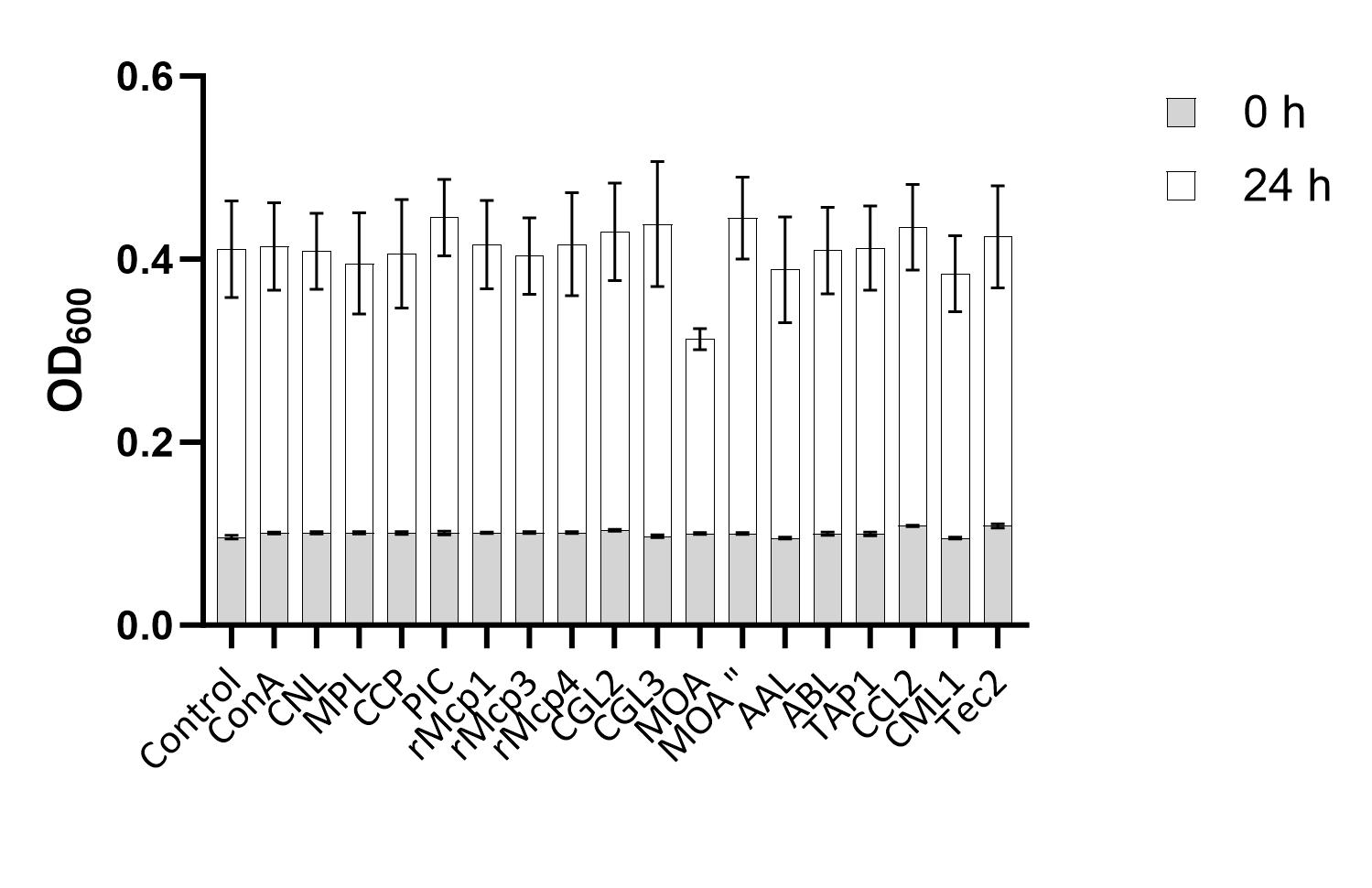


**Supplementary figure 2.** Lectins had no inhibitory effects on *Campylobacter jejuni* growth. Optical density at 600 nm (OD_600_) at 0 h and after 24 h of incubation at 42 °C. Working lectin concentrations of 250 µg/mL had no effect on *C. jejuni* growth, except for MOA. Thus, the concentration of MOA was decreased to 125 µg/mL (MOA”) Abbreviations are defined in Table 1

**Supplementary figure** **3** Correlation analysis using Pearson's method was performed for the data obtained in Supplementary table 1. The results show a strong correlation between the effects of selected lectins and protease inhibitors on the modulation of *Campylobacter jejuni* adhesion to collagen type I and mucin type II (Pearson correlation coefficient value of 0.59 and *P-*value of 0.01)
